# Propofol-Induced Fasciculations in a Patient With Obstructive Sleep Apnea: A Case Report

**DOI:** 10.7759/cureus.75559

**Published:** 2024-12-11

**Authors:** Jaden Y Fang, Tomohiro Yamamoto, Satoshi Yamamoto

**Affiliations:** 1 Anesthesiology, University of Texas Medical Branch, Galveston, USA; 2 Department of Medicine, Gunma University School of Medicine, Maebashi, JPN

**Keywords:** hypoglossal nerve, hypoglossal nerve stimulator insertion, myoclonus, obstructive sleep apnea (osa), propofol-induced myoclonus

## Abstract

We report a case of a 39-year-old male patient who developed propofol-induced fasciculations during the induction of general anesthesia. The patient had a history of moderate obstructive sleep apnea and was intolerant to continuous positive airway pressure therapy. He subsequently underwent the insertion of a hypoglossal nerve stimulator as a viable surgical intervention. The patient had a drug-induced sleep endoscopy that showed a 100% obstruction at the velum and the oropharynx, mainly in the anteroposterior and lateral directions. The patient experienced a smooth induction and emergence from general anesthesia, except for a brief episode of myoclonus-like movement in the bilateral upper extremities after propofol administration. The patient recovered well and reported an improvement in his sleep quality and daytime symptoms.

## Introduction

Propofol is a widely used intravenous anesthetic agent known for its rapid onset and short duration of action. However, one of its less common side effects is the induction of fasciculations, i.e., small, localized, involuntary muscle twitches or contractions. These involuntary, brief, shock-like muscle contractions can occur during the induction phase of anesthesia and are generally benign and self-limiting. The incidence of propofol-induced fasciculations is unknown. While the exact mechanism behind these movements is not fully understood, several theories have been proposed, including gamma-aminobutyric acid (GABA) receptor activation, N-methyl-D-aspartate (NMDA) receptor inhibition, and subcortical disinhibition [1,2]. Understanding and recognizing propofol-induced fasciculations are important for anesthesiologists to differentiate them from other causes of perioperative myoclonus, enabling accurate evaluation of underlying causes and enhancing patient care.

## Case presentation

We present the case of a 39-year-old male patient with a history of moderate obstructive sleep apnea (OSA), hypertension, and obesity, but no known history of seizures, myoclonus, and abnormal exercise tolerance. He was referred to our sleep clinic for the evaluation of continuous positive airway pressure (CPAP) intolerance. The patient had been diagnosed with OSA two years earlier, based on a polysomnography that showed an Apnea-Hypopnea Index (AHI) of 21 events per hour, a lowest oxygen saturation of 82%, and a snoring index of 258 events per hour. The patient was prescribed CPAP therapy with a pressure of 10 cm H2O, but he reported poor adherence and discomfort with the mask. He also complained of persistent daytime sleepiness, fatigue, nocturnal snoring, and poor concentration. He denied any history of smoking, alcohol use, or drug abuse. His medications included lisinopril 10 mg daily and multivitamins. His family history was significant for OSA and hypertension in his father.

On physical examination, the patient was alert and oriented, with a blood pressure of 140/90 mmHg, a pulse of 80 beats per minute, a respiratory rate of 16 breaths per minute, and a temperature of 36.5°C. His height was 1.829 m (6' 0.01"), his weight was 98.5 kg (217 lb 2.5 oz), and his body mass index (BMI) was 29.44 kg/m². His general appearance was normal, with no signs of distress. His skin was intact, with no rashes, abscesses, or masses. His head and neck examination showed no lymphadenopathy, thyromegaly, or tracheal deviation. Neck circumference was within normal limits. His temporomandibular joint examination showed no clicking or popping on opening, no deviation on opening, and a maximum incisal opening of 40 mm. His throat examination showed normal mucosa, uvula midline and mobile, and normal gag reflex. His oral mucosa examination showed no white lesions, red lesions, swelling, or ulcerations. His tongue and floor of the mouth examination showed no swellings, ulcerations, or tenderness to palpation, and a normal gag reflex.

The preoperative evaluation was unremarkable. The patient was classified as an American Society of Anesthesiologists Physical Status II [3]. The airway assessment revealed a Mallampati score of II, thyromental distance exceeding five centimeters, normal neck mobility, normal facial features, and no significant dental issues.

The patient underwent a drug-induced sleep endoscopy procedure, which showed a 100% obstruction at the velum, with 75% in the anteroposterior direction and 25% in the lateral direction, and a 100% obstruction at the oropharynx mainly in the lateral direction. The patient was deemed a good candidate for hypoglossal nerve stimulator (HNS) insertion, and following discussions of risks, benefits, and alternatives of the procedure he consented to the surgery and was scheduled for it.

The surgery was planned under general anesthesia. The patient was premedicated with midazolam 2 mg and fentanyl 50 mcg, both given intravenously. He was monitored with standard monitors, including electrocardiogram, pulse oximetry, noninvasive blood pressure, and bispectral index. He received oxygen via a facemask at six liters/min. He was induced with propofol at 200 mg intravenously. After propofol administration, he developed a brief episode of involuntary muscle movement in the bilateral upper extremities that lasted for three seconds (Video 1). The movement observed before mask ventilation was limited to the upper extremities, asymmetrical in nature, and occurred without any associated hemodynamic changes, oxygen desaturation, or electroencephalographic abnormalities. The mask ventilation was not affected. The movement resolved spontaneously, and the patient was intubated without difficulty following the administration of succinylcholine at 100 mg.

**Video 1:** Propofol-induced fasciculations during hypoglossal nerve stimulator insertion

Endotracheal intubation was then performed using direct laryngoscopy with a Macintosh size 4 blade (IntuBrite, Salter Labs, Arvin, California, USA). Anesthesia was maintained with 2% sevoflurane and 0.1 µg/kg/min remifentanil in conjunction with volume-controlled ventilation. The surgery was performed by the otolaryngology team, who implanted the pulse generator in the right upper chest, with the stimulation lead in the right submandibular region, and the sensing lead in the right intercostal muscles. The surgery was uneventful and lasted for two hours. The patient was extubated and transferred to the post-anesthesia care unit where he recovered well. He had no recurrence of myoclonus-like movement and no postoperative complications. He was discharged the next day and instructed to follow up with the sleep clinic for device activation and titration.

## Discussion

Propofol is a widely used intravenous anesthetic known for its rapid onset and short duration of action. However, it can also induce muscle fasciculations, also called involuntary muscle twitches [4,5]. Propofol-induced fasciculation is a rare phenomenon. Its pathophysiology is not fully understood, but several mechanisms have been proposed, such as gamma-aminobutyric acid (GABA) receptor activation, N-methyl-D-aspartate (NMDA) receptor inhibition, opioid receptor modulation, subcortical disinhibition, and genetic factors [1,2]. While propofol-induced fasciculation is usually benign and self-limiting, it can be distressing for patients and anesthesiologists since it mimics seizure activity or indicates an underlying neurological disorder. Therefore, it is important to differentiate propofol-induced fasciculation from other causes of perioperative myoclonus, such as hypoxia, electrolyte imbalance, metabolic acidosis, hepatic encephalopathy, uremic encephalopathy, drug toxicity, or withdrawal [6-8]. Furthermore, meticulous observation is essential to differentiate the fine muscle twitches associated with fasciculations from the larger, escape-like movements characteristic of an escaping reaction, or the rigidity caused by fentanyl-induced chest wall stiffness, thereby ensuring an accurate diagnosis.

In contrast to propofol-induced fasciculation, the risk factors for propofol-induced myoclonus include being young and male (aged 15 to 60), anxiety, opioid premedication, high-dose propofol, and rapid injection [9]. While obstructive sleep apnea syndrome (OSAS) is not directly linked to propofol-induced myoclonus, individuals with OSAS are more prone to sedative-related complications, including myoclonus, i.e., sudden, large, involuntary muscle jerks. The prevention and treatment of propofol-induced myoclonus are not well established, but some strategies have been suggested. These include low-dose propofol, slow injection, or pretreatment with lidocaine, midazolam, dexmedetomidine, or magnesium sulfate [10,11]. In our case, the patient’s age, gender, and opioid premedication posed some risks for propofol-induced myoclonus. He also received a dose of propofol (200 mg) for induction which might have contributed to the occurrence of the myoclonus-like movement. However, he had no other signs of seizure activity, such as hemodynamic changes, oxygen desaturation, or electroencephalographic changes, and had a smooth emergence and recovery. Therefore, we concluded that the myoclonus-like movement was fasciculation likely due to propofol and not related to any other cause.

## Conclusions

We reported a case of a 39-year-old male patient with moderate OSA and CPAP intolerance who developed propofol-induced fasciculation while undergoing HNS insertion as a surgical option. The patient had a smooth induction and emergence from general anesthesia, except for a brief episode of fasciculation in the bilateral upper extremities after propofol administration. The movement was benign and self-limiting and did not affect the outcome of the surgery. The patient recovered well and reported improvement in his sleep quality and daytime symptoms. Given the asymmetrical, fine, involuntary movements in the upper extremities, we present a case of the rare and typically benign phenomenon of propofol-induced fasciculation, which should be carefully distinguished from other causes of perioperative myoclonus.
